# Serum Biomarkers for the Detection of Cardiac Toxicity after Chemotherapy and Radiation Therapy in Breast Cancer Patients

**DOI:** 10.3389/fonc.2014.00277

**Published:** 2014-10-09

**Authors:** Sibo Tian, Kim M. Hirshfield, Salma K. Jabbour, Deborah Toppmeyer, Bruce G. Haffty, Atif J. Khan, Sharad Goyal

**Affiliations:** ^1^Department of Radiation Oncology, Rutgers Cancer Institute of New Jersey and Rutgers Robert Wood Johnson Medical School, New Brunswick, NJ, USA; ^2^Division of Medical Oncology, Rutgers Cancer Institute of New Jersey and Rutgers Robert Wood Johnson Medical School, New Brunswick, NJ, USA

**Keywords:** breast cancer, cardiac biomarkers, chemotherapy, radiation therapy, cardiotoxicity

## Abstract

Multi-modality cancer treatments that include chemotherapy, radiation therapy, and targeted agents are highly effective therapies. Their use, especially in combination, is limited by the risk of significant cardiac toxicity. The current paradigm for minimizing cardiac morbidity, based on serial cardiac function monitoring, is suboptimal. An alternative approach based on biomarker testing, has emerged as a promising adjunct and a potential substitute to routine echocardiography. Biomarkers, most prominently cardiac troponins and natriuretic peptides, have been evaluated for their ability to describe the risk of potential cardiac dysfunction in clinically asymptomatic patients. Early rises in cardiac troponin concentrations have consistently predicted the risk and severity of significant cardiac events in patients treated with anthracycline-based chemotherapy. Biomarkers represent a novel, efficient, and robust clinical decision tool for the management of cancer therapy-induced cardiotoxicity. This article aims to review the clinical evidence that supports the use of established biomarkers such as cardiac troponins and natriuretic peptides, as well as emerging data on proposed biomarkers.

## Introduction

Due to earlier detection and highly effective multi-modality treatments, cancer has become a largely curable disease and a chronic illness. There were an estimated 11.7 million cancer survivors in 2007, a number that has grown from 3.0 million in 1970, to 9.8 million in 2001 (1). The Centers for Disease Control estimated in 2007 that 64.8% of cancer survivors had lived at least 5 years past their initial diagnosis, and approximately 60% of survivors were at least 65 years old. Because of the now-chronic nature of malignant diseases, and the age composition of the survivors, the cardiac side effects of cancer treatments must be heeded. Cytotoxic chemotherapies such as doxorubicin, targeted therapies including trastuzumab, and radiotherapy have all been implicated as risk factors for subsequent cardiac disease. The timing of cardiac toxicity can vary from acutely during treatment, to chronically months after treatment completion. The most clinically significant endpoint is impaired left ventricular ejection fraction (LVEF) and ensuing symptomatic heart failure. The current standard of detection is by serial echocardiography, a resource-intensive test whose accuracy is operator-dependent. Biomarkers on the other hand, can be tested at closer intervals given its low-cost approach; and its accuracy is independent of operator skill. Most importantly, biomarkers have demonstrated the ability to predict cardiotoxicity before it becomes clinically apparent. The use of cardiac biomarker in specific settings have been reviewed several times, and most recently in 2011 (2–6). However, the role of biomarkers is continually redefined by ongoing investigations. The purpose of this review is to provide a comprehensive assessment of the evidence on cardiac troponins and natriuretic peptides as biomarkers of cardiac toxicity. Results for other proposed biomarkers, including heart-type fatty acid-binding protein (H-FABP), glycogen phosphorylase isoenzyme BB (GPBB), C-reactive protein (CRP), myeloperoxidase (MPO), and nitric oxide (NO) will also be examined.

## Cardiac Toxicity after Cancer Treatment

Anthracyclines (AC), either used alone, or in combination with other chemotherapy agents, are widely used agents for the treatment of breast cancer (7). However, their use has been limited by significant cardiotoxicity (8). AC-induced injury has been described as “type I” cardiotoxicity, a dose-dependent, progressive, and generally irreversible type of toxicity (9). Its mechanism is based on oxidative damage, mediated by reactive oxygen species, and leads to necrosis and apoptosis (10). Risk of developing AC-induced cardiotoxicity varies between individuals, and even low doses have led to clinical cardiac dysfunction for certain patient subsets (11). Risk factors for developing AC-induced cardiotoxicity include cumulative dose, age, female gender, exposure to cardiotoxic agents, prior AC chemotherapy, and mediastinal radiation. The clinical manifestations of AC-associated cardiotoxicity range from left ventricular dysfunction to progressive cardiomyopathy. Doxorubicin administration is generally limited to a cumulative dose of 600 mg/m^2^ in patients without underlying cardiac morbidity (12).

The pediatric population is particularly susceptible to AC-induced cardiomyopathy; and there is likely no safe dose in children (13). The incidence of cardiotoxicity after AC treatment in childhood is similarly dose-dependent: 11, 23, 47, and 100% suffered from cardiac complications after being treated with <400, 400–599, 600–799, and >800 mg/m^2^ of AC-based chemotherapy (14, 15). Treatment with ACs has long-term implications. Survivors of pediatric cancers are 8.2 times more likely to die from cardiac causes than the general population, and 15 times more likely to experience heart failure, with some eventually requiring heart transplants (16–18).

About 25–30% of breast cancers overexpress the cell surface receptor HER2. These malignancies are typically more aggressive, with enhanced proliferation and metastatic potential, and are associated with poor prognosis (19). Trastuzumab (Herceptin) is a monoclonal antibody that binds to the extracellular domain of the HER2 protein. Its efficacy in the adjuvant setting has been investigated in numerous clinical trials. A meta-analysis demonstrated reduction in mortality, recurrence and metastases rates, and improved disease-free survival with trastuzumab (20). Trastuzumab, though generally well tolerated, is associated with an infrequent but clinically significant risk of long-term cardiotoxicity. Unlike AC-induced cardiac injury, trastuzumab is described as “type II” cardiotoxicity. The risk of damage is dose-independent, generally reversible with discontinuation, and causes minimal ultrastructural changes (21–23). The risk of developing trastuzumab-induced heart failure has been reported as 2–4% when given alone, but as high as 27% when administered in conjunction with ACs (24, 25). With the advent of newer HER2-directed therapies, additional consideration will need to be given to long-term cardiac side effects associated with their use. Clinical trials have reported fewer grade three or four cardiac toxicity with lapatinib, pertuzumab, trastuzumab emtansine (T-DM1), or neratinib in comparison to trastuzumab (26–34). As other HER2-targeted agents are under development or evaluation for combinatorial therapy, cardiotoxicity will remain a topic of interest.

Radiation therapy (RT) is major component cancer treatment; and adjuvant radiotherapy for breast cancer reduces the risk of local recurrences and mortality (35). However, mediastinal irradiation has been linked to increased cardiotoxicity, via micro- and macrovascular damage (36, 37). A surveillance, epidemiology, and end results (SEER) analysis of 15,165 breast cancer patients found that of those who died more than 10 years after radiotherapy, 42% died from recurrent breast cancer, while 22% died from heart disease (38). The severity of cardiac injury is related to the radiation dose absorbed by the heart, and mean heart dose is typically higher when RT is to employed to treat left-sided breast cancer. The SEER study found patients with left-sided cancers had a 44% increased risk of cardiac mortality. Based on several randomized studies, the relative risk for significant cardiac events ranges 1.2–3.5 after RT (39). As RT is often combined with chemotherapy, cardiac irradiation has been described repeatedly as an additional risk factor for AC-induced cardiotoxicity (40, 41). Though data are still maturing on the cardiac risks of radiotherapy delivered concurrently with trastuzumab, an analysis of the NCCTG N9831 trial showed no additional cardiotoxicity with RT (42). Advances in radiation delivery technology, such as conformal radiation, which limit the amount of radiation absorbed by the myocardium, have proven useful in reducing the burden of radiation-induced cardiac morbidity (38, 43, 44). Regardless, prior mediastinal irradiation remains a significant cause of excessive mortality.

## Detection of Cardiac Dysfunction

Clinically detectable cardiotoxicity is generally preceded by an interval of subclinical cardiac dysfunction. The ability to assess the risk of potential cardiac impairment has three major implications. Risk stratification provides an opportunity to modify ongoing treatment, alter the frequency of subsequent surveillance, and to provide direct interventions to reduce the risk of cardiotoxicity. For these reasons, techniques for early and reliable detection of clinically silent cardiotoxicity have been widely studied. Though several methods been explored, the optimal approach and timing of monitoring cardiac function remains an area of active investigation.

Serial endomyocardial biopsies, though considered the gold standard are invasive and impractical for routine screening purposes (45). The most prevalent screening method is based on measuring LVEF before, during, and after chemotherapy with conventional 2-D transthoracic echocardiography (TTE) (46). Monitoring with multiple-gated acquisition (MUGA) radionuclide angiography has also been recommended on the basis of improved accuracy (47). Because 2-D TTEs can be often limited by operator skill, and inherently less reproducible, efforts have been directed toward increasing its precision with refinements such as 3-D echocardiography, strain and strain rate measurements, and cardiac magnetic resonance (48–51). LVEF measurements based on cardiac imaging lack the sensitivity to detect early subclinical cardiotoxicity, and as a corollary, the ability to predict future declines in cardiac function (52, 53). Detectable changes in LVEF usually coexist with significant functional impairment, at which point the ability to regain normal cardiac function becomes limited. Thus, the traditional approach for detecting subclinical signs of cardiotoxicity is suboptimal and there remains a need to effectively identifying patients who are at risk of developing serious cardiac complications after chemotherapy or RT.

Over the past 15 years, serum molecules, such as cardiac troponins and natriuretic peptides, have been evaluated for their role as biomarkers of cardiac toxicity in the oncology setting. The ability of these biomarkers to identify patients with potential cardiac morbidity has been investigated in adult and pediatric populations, after chemotherapy, radiation, and targeted therapies. Biomarkers represent a non-invasive, resource-efficient, and robust approach to risk-stratify patients who have undergone cardiotoxic treatments.

## Cardiac Troponins

Cardiac troponin I (TnI) and cardiac troponin T (TnT) are two highly sensitive and specific biomarkers of cardiac damage. They are two tissue-specific isoforms of proteins that constitute the contractile apparatus in cardiac muscle. Since 2000, the European Society of Cardiology and the American Cardiac College of Cardiology have recognized cardiac troponins for their role in the diagnosis of acute myocardial infarctions (54, 55). Cardiac troponins have been useful in quantifying the extent of acute cardiomyocyte injury in many other clinic settings, including heart failure, pulmonary embolism, stroke, sepsis, and drug-induced cardiotoxicity (56–58). Notably, because cardiac troponin concentrations have been linked to the severity of myocyte injury and subsequent clinical outcomes, troponins have become a tool for risk stratification.

The validity of using cardiac troponins in detecting chemotherapy-induced cardiotoxicity was demonstrated in an early animal study that linked TnT elevations to histologic evidence of cardiac damage (59). Using spontaneously hypertensive rats treated with increased higher doses of doxorubicin, TnT and Billingham cardiomyopathy scores (based on number of myocytes showing myofibrillar loss and cytoplasmic vacuolization) were both related to the cumulative dose of doxorubicin. Cardiac troponins have consistently demonstrated clinical value in predicting subsequent cardiotoxicity after high-dose chemotherapy (HDC), irrespective of cancer type. This result is based on four major experiences that enrolled approximately 200–700 patients each (Table 1) (60–63). Cardiac troponins are sensitive and specific markers in predicting the development, and severity of, subsequent ventricular dysfunction. The largest study, involving 703 patients (46% breast cancer) with advanced cancers treated with HDC (62). TnI was assayed immediately and 1 month after chemotherapy, while cardiac function was measured by LVEF at baseline, and 1, 2, 6, and 12 months after completing chemotherapy. Thirty percent (208) of patients demonstrated immediate TnI elevations, and 30% of that subset showed elevated TnI on repeat testing at 1 month. Maximal LVEF reduction was predicted by both persistent (*r* = 0.92, *p* < 0.001), and early (*r* = 0.78, *p* < 0.001) troponin elevations. Most importantly, TnI proved to be a biomarker with clinical implications, and not simply a proxy for imaging-based measures. Forty-four percent of patients with persistent TnI elevations developed symptomatic heart failure, compared to 12% in the early positive group, and 0.2% in the TnI negative population. Troponin positivity over 0.08 ng/m^2^ predicted future cardiac events with a positive predictive value (PPV) of 84% and negative predictive value (NPV) of 99%. TnI’s high NPV has been a recurrent theme seen in many studies.

**Table 1:** **Role of cardiac troponins in the evaluation of chemotherapy and radiation-induced cardiotoxicity**.

Reference	Population	N	Treatment	Tn type	Cutoff	Troponin evaluations	Results and conclusions
Hugh-Davies et al. (64)	Breast cancer	50	ACs and RT	T	0.1 ng/ml	Pre- and post-treatment	No change in TnT after 45–46 Gy delivered to the whole breast
Lipshultz et al. (65)	ALL	15	ACs	T	0.03 ng/ml	Baseline, and 1–3 days after each cycle	Correlation between TnT and LV end-diastolic dimension and wall thickness
Herman et al. (59)	Animal study	37	ACs	T		Before, and 1 week after chemotherapy	TnT and histological myocardial changes in both related to cumulative doxorubicin dose
Cardinale et al. (60)	Various	204	HDC	I	0.5 ng/ml	Before, and 0, 12, 24, 36, and 72 h after every cycle	Elevated TnI during treatment predicted for LVEF decline
Cardinale et al. (61)	Breast cancer	211	HDC and RT	I	0.5 ng/ml	Before, and 0, 12, 24, 36, and 72 h after every cycle	Correlation between max TnI, number of TnI positive assays, and max LVEF reduction
Auner et al. (66)	Hematologic malignancies	78	ACs	T	0.03 ng/ml	Within 48 h of treatment start, then every 48 h during treatment	Correlation between TnT increase and median LVEF decline
Sandri et al. (63)	Various	179	HDC	I	0.08 ng/ml	Before, and 0, 12, 24, 36, and 72 h after every cycle	TnI increase predicted subsequent LVEF decline
Cardinale et al. (62)	Various	703	HDC	I	0.08 ng/ml	Before, and 0, 12, 24, 36, and 72 h after every cycle, and 1 month after treatment	Persistent TnI positivity predicted for subsequent LVEF decline
Kismet et al. (67)	Pediatric solid cancers	24	ACs	T	0.01 ng/ml	With imaging, >1 month after chemo	No relationship between TnT and echocardiographic abnormalities
Lipshultz et al. (68)	ALL	76	ACs	T	0.01 ng/ml	Throughout chemotherapy	TnT persistently increased during treatment, and predicted for cardioprotective response
Kilickap et al. (69)	Various	41	ACs	T	0.01 ng/ml	Baseline, after first and last cycle	Correlation between TnT increase and diastolic dysfunction (E/A ratio)
Perik et al. (70)	Breast cancer	17	ACs and T	I	0.1 g/l	Before, and throughout T therapy	No TnI elevations in 15/16 patients
Dodos et al. (71)	Various	100	ACs	T	0.1 ng/ml	After first dose, last dose, and 1, 6, 12 months after last dose	No TnT elevations detected
Kozak et al. (72)	Lung and esophageal CA	30	ChemoRT	T		Baseline, 2 weeks after start of treatment and after	TnT undetectable in 29/30 patients
Cil et al. (73)	Breast cancer	33	ACs	I		Before and after chemotherapy	No correlation between TnI and LVEF decline
Mavinkurve-Groothuis et al. (74)	Various pediatric	122	ACs	T	0.01 ng/ml	Once, with imaging	No patients with elevated TnT levels
Cardinale et al. (75)	Breast cancer	251	ACs and T	I	0.08 ng/ml	Before T, every 3 months during treatment, 1 year after start, every 6 months	Elevated TnI values are an independent predictor of cardiotoxicity, and LVEF recovery
Nellessen et al. (76)	Lung and breast CA	23	RT	I	0.03 ng/ml	Before RT, every week during RT for 4–6 weeks	Log-transformed TnI increased during treatment
Fallah-Rad et al. (51)	Breast cancer	42	ACs and T	T		Before chemotherapy, before T, and 3, 6, 9, and 12 months after start of T	No change in TnT values over time
Feola et al. (77)	Breast cancer	53	ACs	I	0.03 ng/ml	Baseline, after 1 month, 1, and 2 years	TnI concentrations elevated at 1 month, then returned to normal
Goel et al. (78)	Breast cancer	36	ACs and T	I	0.20 ng/ml	Baseline, before and 24 h after T	No elevated TnI values throughout
Morris et al. (79)	Breast cancer	95	ACs and T	I	0.04–0.06 ng/ml	Every 2 weeks during treatment, then at 6, 9, and 18 months	Elevated TnI values preceded maximal LVEF decline, but no relationship with max LVEF decline
Romano et al. (80)	Breast cancer	92	ACs	I	5 or 0.08 ng/ml (age ≤50 or >50)	Every 2 weeks during treatment, then at 3, 6, and 12 months	No correlation between TnI change and subsequent LV impairment
Sawaya et al. (81)	Breast cancer	43	ACs and T	I	0.015 ng/ml	Baseline, 3 and 6 months after chemotherapy	Elevated TnI at 3 months predicted for cardiotoxicity within 6 months
D’Errico et al. (82)	Breast cancer	60	ChemoRT	I	0.07 ng/ml	Before, and after RT	No elevated TnI concentrations
Garrone et al. (83)	Breast cancer	50	ACs	I	0.03 ng/ml	Baseline, 5, 16, and 28 months after	TnI kinetics correlated with LVEF decline
Lipshultz et al. (84)	ALL	156	ACs	T	0.01 ng/ml	Before, and daily during induction, and after treatment	Lower incidence of detectable TnT during treatment with dexrazoxane
Onitilo et al. (85)	Breast cancer	54	Taxanes and T	I	0.1 ng/ml	Baseline, and every 3 weeks during treatment	TnI undetectable throughout
Sawaya et al. (86)	Breast cancer	81	ACs and T	I	30 pg/ml	Before, every 3 months during, and after T treatment	Elevated TnI values at end of treatment predictive of subsequent cardiotoxicity
Sherief et al. (87)	Acute leukemias	50	ACs	T	0.01 ng/ml	Once, with imaging	No elevated TnT values
Erven et al. (88)	Breast cancer	72	RT	I	0.13 ng/ml	Before and after RT	Higher TnI values in L-sided breast patients
Ky et al. (89)	Breast cancer	78	ACs and T	I	121.8 ng/ml	Baseline, 3 and 6 months after start of chemotherapy	Interval change in TnI predicted cardiotoxicity

*Tn, troponin; AC, anthracycline; RT, radiation therapy; HDC, high-dose chemotherapy; T, trastuzumab; LVEF, left ventricular ejection fraction; ALL, acute lymphoblastic leukemia*.

Left ventricular ejection fraction compromises with high-dose chemo can be evident as early as the first month, and was typically followed by progressive deterioration over the next year (61). In addition, smaller studies have found substantial relationships between troponin velocity during early follow-up and decreased LVEF (83). Elevated troponins have been implicated in predicting diastolic dysfunction via parameters such as E/A ratio in particular patient subsets treated with AC (69). Conversely, the role for troponin in low and moderate chemotherapy doses in unclear, as evaluated in a study with 100 patients treated with AC (median cumulative dose 226.1 mg/m^2^) (71). Even with TnT being assayed at five intervals from the first dose of chemotherapy to 12 months after its completion, no patient had recorded TnT values above the 0.1-ng/ml threshold. Of those who showed TnT rises after treatment, the majority reported normal LVEF and E/A ratio values just 1 year after completing chemotherapy.

Notably, cardiac troponins have been key in facilitating the evaluation of cardioprotective agents in two prospective randomized trials (68, 84). Both randomized children diagnosed with acute lymphoblastic leukemia (ALL) to doxorubicin with or without dexrazoxane, a free radical scavenger. In both studies, dexrazoxane drastically reduced the incidence of above-threshold values TnT during treatment. In the more recent experience, TnI levels during the first 90 days of treatment predicted lower LV mass and LV end-diastolic posterior wall thickness 4 years later (84).

Reports of troponin as a prognostic tool in asymptomatic survivors of childhood cancers have been largely disappointing. An early study of children treated with doxorubicin found the magnitude of TnT elevation after the first dose of chemotherapy predicted for the risk of subsequent echocardiographic abnormalities, including LV dilation (*r* = 0.8, *p* = 0.003), and LV wall thinning (*r* = 0.61, *p* = 0.04) 9 months later (65). The timing of injury markers supported the hypothesis that AC-induced injury can begin as early as the first dose, and is driven by continuous oxidative stress rather than acute necrosis. However, numerous studies discovered either no above-threshold troponin values, or lacked substantial relation with late-onset cardiac toxicity in survivors of childhood malignancies (67, 74, 87, 90).

In parallel with the growing usage of adjuvant trastuzumab in patients with HER2 overexpressing or amplified breast cancer, several large-scale studies have found a well-defined relationship between either troponin value or its interval change and tratuzumab-induced cardiac dysfunction. Cardinale et al. provided the earliest evidence cardiac troponin values can stratify patients on risk of developing trastuzumab-induced cardiotoxicity, based on 251 breast cancer patients who were followed for a median of 14 months after completion of trastuzumab treatment (75). Thereafter, systolic function (LVEF) was evaluated via echocardiography at baseline, every 3 months during trastuzumab treatment and the first year of follow-up, and then every 6 months. Forty-two (17%) patients developed cardiac review and evaluation committee (CREC)-defined cardiac dysfunction; however, those with above-threshold TnI concentrations were at significantly higher risk for cardiotoxicity (62 vs. 5%, *p* < 0.001). Moreover, TnI positivity was the strongest independent predictor of cardiotoxicity (HR = 17.6, *p* < 0.001) and persistent LVEF impairment (HR 2.33, *p* < 0.001). Troponin positivity predicted LVEF recovery with a PPV of 65% and NPV of 100%. This suggested that negative TnI measurements during treatment can be used to assign a lower risk status to select patients who are less likely to benefit from cardiac screening at routine intervals.

With regard to the timing of troponin rises with trastuzumab treatment, Morris et al. found peak TnI elevations peaked occurred approximately 2 months and four after dose-dense AC-based chemotherapy (79). Importantly, it preceded maximum LVEF decline by 4 months. Two studies by Sawaya et al. supported these results. Both examined TnI in patients who were treated with AC and trastuzumab sequentially. They first found that elevated high-sensitivity (hs)TnT measurements 3 months after chemotherapy was an independent predictor of cardiac toxicity at 6 months (81). The follow-up study combined circulating biomarkers with echocardiographic measures to refine their predictive model. Using an ultrasensitive troponin assay that established 30 pg/ml as the cutoff concentration, they found TnI alone predicted subsequent cardiotoxicity with PPV of 44% and NPV of 77% (86). Adding peak systolic longitudinal strain of <19% improved the specificity of the model, yielding a PPV of 67% and NPV of 77%. Interestingly, baseline LVEF at the time of AC completion did not predict for future cardiotoxicity. Though the majority of studies evaluating troponins in trastuzumab-induced cardiac damage have demonstrated its usefulness, several experiences have been negative (51, 77–79).

Despite abundant literature on radiation-induced cardiac injury, troponins have yet to demonstrate any clinical utility. Studies in which considerable numbers of patients were treated with RT as a single modality are relatively scarce. Of those that try to isolate the effect of radiotherapy, none have been able draw clinically valuable conclusions regarding the value of troponin in predicting radiation-induced cardiotoxicity (64, 72, 82). In fact, of four studies that included patients with breast, lung, and esophageal cancer, only one saw significantly elevated TnI concentrations after RT (88).

## Natriuretic Peptides

Natriuretic peptides, such atrial natriuretic peptide (ANP), brain natriuretic peptide (BNP), and its amino-terminal component (NT-proBNP) have been widely investigated and used in acute and chronic heart failure for diagnosis and prognosis. In response to increased wall stress, BNP is synthesized by ventricular cardiomyocytes as a 134-amino acid (aa) pre-pro peptide, which is then cleaved into a 108-aa precursor molecule (proBNP). Upon release, proBNP is cleaved into an inactive N-terminal component (NT-proBNP) and the 32-residue active hormone BNP. To counteract volume overload, biological actions of BNP include natriuresis, vasodilation, and suppression of sympathetic activity (91). Chronic elevations in BNP reflect increased LV wall stress diastolic pressure, and volume overload (92, 93). Moreover, NT-proBNP concentrations have been related to LVEF values and the severity of hearth failure (94). Thus, using natriuretic peptides to risk-stratify patients with potential cardiotoxicity would intuitively be an attractive strategy, as they represent hemodynamic aberrancy and ventricular remodeling, and can appear prior to symptomatic heart failure and LVEF decline (95).

A large number of studies have described significant BNP and NT-proBNP elevations with doxorubicin, epirubicin, trastuzumab, and thoracic irradiation, either alone in combination therapy, though substantially fewer have found clinical relevant relationships (Table 2). One early study that established the predictive value of NT-proBNP examined its role in patients with various advanced malignancies treated with high-dose AC-based chemotherapy (63). Sandri et al. measured NT-proBNP at baseline, and then at five time points within 72 h of completing each treatment cycle. Persistent NT-proBNP measurements predicted for the development of cardiac dysfunction at 12 months when quantified by three LV diastolic indices. The predictive value of early NT-proBNP rises was also seen with a cohort of breast cancer patients with doxorubicin to a cumulative dose of 300 mg/m^2^ (80). Post-chemotherapy NT-proBNP increases were related to subsequent LVEF decline (*r* = 0.7, *p* ≤ 0.001). An ROC analysis using a cutoff of >36% NT-proBNP increase from baseline to peak predicted LV impairment at 12 months after therapy with 79.2% sensitivity and specificity. Similar correlations between NT-proBNP elevations and LVEF values in the setting of breast cancer treated with moderate dose epirubicin and non-Hodgkin lymphoma patients after six cycles of CHOP chemotherapy (96, 97).

**Table 2:** **Role of natriuretic peptides in the evaluation of chemotherapy and radiation-induced cardiotoxicity**.

Reference	Population	N	Treatment	BNP type	Cutoff	BNP evaluations	Results and conclusions
Meinardi et al. (98)	Breast cancer	39	ACs and RT	BNP	10 pmol/l	Baseline, 1 month, and 1 year after chemotherapy	BNP increased as early as 1 month after chemo; no correlation with LVEF decline
Nousiainen et al. (99)	Non-Hodgkin lymphoma	28	CHOP	BNP	227 pmol/l	Baseline, after every cycle, and 4 weeks after last cycle	Correlation between BNP increases and parameters of diastolic function (FS and PFR)
Daugaard et al. (100)	Various	107	ACs	BNP		Before, and at various points during treatment	BNP correlation with decreased LVEF, but baseline and BNP change could not predict LVEF decline
Perik et al. (101)	Breast cancer	54	ACs and RT	NT-proBNP	10 pmol/l	Median 2.7 and 6.5 years after chemotherapy	BNP increased with time and was related to dose; cardiotoxic effects develop over years
Sandri et al. (102)	Various	52	HDC	NT-proBNP	153 ng/l (M ≤50), 227 ng/l (M >50), 88 ng/l (F ≤50), 334 ng/l (F >50)	Baseline, and 0, 12, 24, 36, and 72 h after each cycle	Persistent NT-proBNP elevation at 72 h predicts later systolic and diastolic dysfunction
Germanakis et al. (103)	Pediatric cancers	19	ACs	NT-proBNP	0.2 pmol/ml	Mean 3.9 years after chemotherapy	Correlation between NT-proBNP and LV mass decrease
Perik et al. (70)	Breast cancer	17	ACs and T	NT-proBNP	125 ng/l	Baseline and throughout T treatment	Higher pre-treatment NT-proBNP values in those who developed HF during treatment
Aggarwal et al. (104)	Pediatric cancers	63	ACs	BNP		Once, >1 year after treatment completion	Higher BNP in patients with late cardiac dysfunction by ECHO
Ekstein et al. (105)	Pediatric cancers	23	ACs	NT-proBNP	350 pg/ml	Before and after each AC dose	Dose-related increase in BNP from baseline seen after first AC dose
Jingu et al. (106)	Esophageal cancer	197	RT	BNP		Before, <1 month, 1–2, 3–8, 9–24, and >24 months after RT	Increased BNP over time and in those with abnormal FDG accumulation
Kouloubinis et al. (97)	Breast cancer	40	ACs	NT-proBNP		Before and after chemotherapy	Correlation between NT-proBNP increase and LVEF decline
Dodos et al. (71)	Various	100	ACs	NT-proBNP	153 or 227 ng/l for M ≤50 or >50; 88 or 334 ng/l for F ≤50 or >50	After first dose, last dose, and 1, 6, and 12 months after last dose	No significant increase in NT-proBNP with treatment; cannot replace serial ECHO for monitoring of AC-induced cardiotoxicity
Kozak et al. (72)	Lung and esophageal CA	30	ChemoRT	NT-proBNP		Baseline, after 2 weeks of RT, and after RT end	No change in NT-proBNP during treatment
Cil et al. (73)	Breast cancer	33	ACs	NT-proBNP	110 pg/ml	Before and after chemotherapy	Despite association, pre-chemo NT-proBNP did not predict for later LVEF
ElGhandour et al. (96)	Non-Hodgkin lymphoma	40	CHOP	BNP		Before first cycle and after sixth cycle of chemotherapy	Correlation between BNP values after chemotherapy and LVEF
Mavinkurve-Groothuis et al. (74)	Pediatric cancers	122	ACs	NT-proBNP	10 pmol/l (M), 18 pmol/l (F), age-adjusted in children (107)	Once, with imaging	NT-proBNP levels related to cumulative AC dose
Nellessen et al. (76)	Lung and breast CA	23	RT	NT-proBNP	100 pg/ml	Before RT, every week during RT for 4–6 weeks	Log-transformed NT-proBNP increased during treatment
Fallah-Rad et al. (51)	Breast cancer	42	ACs and T	NT-proBNP		Before chemotherapy, before T, and 3, 6, 9, and 12 months after start of T	No change in NT-proBNP values over time
Feola et al. (77)	Breast cancer	53	ACs	NT-proBNP	5 pg/ml	Baseline, after 1 month, 1, and 2 years	NT-proBNP increased acutely with treatment, and in patients with systolic dysfunction
Goel et al. (78)	Breast cancer	36	ACs and T	NT-proBNP	110 pg/ml (age <75), 589 pg/ml (age >75)	Baseline, before and 24 h after T	No change in NT-proBNP with trastuzumab
Romano et al. (80)	Breast cancer	92	ACs	NT-proBNP	153 pg/ml (age ≤50), 222 pg/ml (age >50)	Every 2 weeks during treatment, then at 3, 6, and 12 months	Interval change in NT-proBNP predicated for LV impairment at 3, 6, and 12 months
Sawaya et al. (81)	Breast cancer	43	ACs and T	NT-proBNP	125 pg/ml	Baseline, 3 and 6 months after chemotherapy	No relation between NT-proBNP levels before and after treatment and LVEF change
D’Errico et al. (82)	Breast cancer	60	ChemoRT	NT-proBNP	125 pg/ml	Before, and after RT	Correlation between NT-proBNP, V_3Gy_ for the heart, $D_{15cm^{2}}∕D_{\mathrm{mean}}$ and $D_{15cm^{3}}∕D_{\mathrm{50}\%}$
Lipshultz et al. (84)	ALL	156	ACs	NT-proBNP	150 pg/ml (age <1), 100 pg/ml (age ≥1)	Before, and daily during induction, and after treatment	Correlation between NT-proBNP and change in LV thickness-to-dimension ratio 4 years later
Mladosievicova et al. (108)	Childhood leukemias	69	ACs	NT-proBNP	105 pg/ml (F), 75 pg/ml (M)	Median 11 years after treatment	Increased NT-proBNP with exposure to ACs
Onitilo et al. (85)	Breast cancer	54	Taxanes and T	BNP	200 pg/ml	Baseline, and every 3 weeks during treatment	No correlation between elevated BNP values and cardiotoxicity
Pongprot et al. (90)	Pediatric cancers	30	ACs	NT-proBNP	Age-adjusted (109)	Once, with imaging	Correlation between NT-pro BNP values and FS and LVEF
Sawaya et al. (86)	Breast cancer	81	ACs and T	NT-proBNP	125 pg/ml	Before, every 3 months during, and after T treatment	NT-proBNP did not change with treatment
Sherief et al. (87)	Acute leukemias	50	ACs	NT-proBNP	Age-adjusted (107)	Once, with imaging	NT-proBNP linked to AC dose and abnormal tissue Doppler imaging parameters
Kittiwarawut et al. (110)	Breast cancer	52	ACs	NT-proBNP	45 pg/ml	Baseline, and end of fourth cycle	Correlation between NT-proBNP and FS
Ky et al. (89)	Breast cancer	78	ACs and T	NT-proBNP		Baseline, 3 and 6 months after start of chemotherapy	No relationship between NT-proBNP values and cardiotoxicity

*BNP, brain natriuretic peptide; NT, N-terminal; AC, anthracycline; RT, radiation therapy; HDC, high-dose chemotherapy; T, trastuzumab; LVEF, left ventricular ejection fraction; HF, heart failure; ALL, acute lymphoblastic leukemia; FS, fractional shortening; PFR, peak filling rate*.

Though early BNP increases have been the focus of many studies for its predictive capabilities, BNP levels can remain elevated up to 2 years after AC-based treatment. This suggests that persistent neurohormonal activation, independent of acute tissue toxicity, is one underlying mechanism of late-onset AC-induced cardiotoxicity (77). BNP monitoring during chemotherapy has also been linked to significant diastolic dysfunction with CHOP. A study by Nousiainen et al. revealed associations between BNP, fractional shortening (FS) (*p* = 0.04), E/A ratio (*p* = 0.006), and trend to significance with LA diameter (*p* = 0.062) (99). Studies involving AC in the adult population have also seen substantial increases in NT-proBNP with no significant interactions with echocardiographic or clinical outcomes (71, 73, 98, 100).

While there has been great interest in validating natriuretic peptides as predictors of cardiotoxicity in the pediatric population, studies in this setting have seen mixed results. NT-proBNP has been shown to be an effect indicator of cardioprotective interventions (84). Specifically, children with ALL were randomized to receive doxorubicin with or without dexrazoxane, an effective free radical scavenger. Lipshultz et al. discovered drastically reduced NT-proBNP concentrations after dexrazoxane treatment (47 vs. 20%, *p* = 0.07). Increased NT-proBNP in the first 90 days of treatment also predicted abnormal LV thickness-to-dimension ratios, suggestive of late-onset LV remodeling. Germanakis et al. evaluated BNP nearly 4 years after AC treatment to find an association between NT-proBNP with LV mass reductions (*p* = 0.003) in asymptomatic survivors (103). Lastly, NT-proBNP concentrations have been consistently identified as a proxy for cumulative AC dose in survivors of childhood cancers (74, 105, 108).

The experience with natriuretic peptides corroborates large-scale studies that have shown the clinic onset of RT-induced cardiotoxicity can occur years after therapy. Significant NT-proBNP elevations have been detected as early as 9 months, and as late as 6.7 years after radiation to the thorax for breast and esophageal cancer (82, 101, 106). In 64 patients with esophageal cancer treated to median dose of 60 Gy, increased NT-proBNP concentrations were found beginning at 9 months (when compared to baseline), and persisted at 24 months after radiotherapy. Additionally, NT-proBNP may be an early indicator of radiation-induced myocardial damage. Substantially, higher natriuretic peptide concentrations were found in subjects with high F-fluorodeoxyglucose (FDG) accumulation on positron emission tomography (PET) corresponding to the irradiated fields (106). Similarly, NT-proBNP has also been linked to cardiac doses in left-sided breast cancer. D’Errico et al. found significant associations between NT-proBNP and V_3Gy_ (volume receiving at least 3 Gy), and two ratios for the heart: D_15cm3_/D_mean_ and D_15cm3_/D_50%_ (where D_mean_ is the mean dose, D_50%_ is the median dose, and $D_{15cm^{3}}$ is the minimum isodose received by 15 cm^3^) (82).

The role of NT-proBNP in predicting trastuzumab-induced cardiac dysfunction has been evaluated in five recent studies. Higher pre-treatment (immediately post-chemotherapy) NT-proBNP concentrations were found in patients with metastatic breast cancer who developed symptomatic heart failure during treatment (*p* = 0.009) (70). The other four failed to find any meaningful relationship between BNP or its interval changes with measures of cardiac function; often no significant changes were found between pre- and post-treatment NT-proBNP concentrations (51, 78, 81, 89). Concerns regarding sufficient follow-up and superimposed AC-induce cardiotoxicity make it unclear whether NT-proBNP has any clinical usefulness in predicting trastuzumab-induced cardiac dysfunction.

## Other Proposed Markers

Heart-type fatty acid-binding protein and glycogen phosphorylase isoenzyme BB have been evaluated jointly as potential biomarkers of cardiac toxicity in several studies. Both GPBB and H-FABP are considered markers of early cardiac injury. GPBB is a cardiac-specific enzyme of glycogenolysis, which provides glucose to cardiac muscle. Because GPBB is released into circulation 2–4 h after myocardial injury, it may be a sensitive, and early marker of acute coronary syndromes. Moreover, GPBB has been found useful for the risk stratification in acute coronary syndromes, as it is an independent predictor of mortality (111). Similarly, H-FABP is a low molecular weight protein normally found in the cytoplasm, but can be detected within 2–3 h after significant myocardial injury (112, 113). In three studies that evaluated GPBB in patients with leukemias and lymphomas, Horacek et al. found approximately 17–21.7% of patients with elevated GPBB concentrations after either AC-based chemotherapy or a preparative regimen for hematopoietic stem cell transplantation (114–116). Based on threshold values of 7.30 μg/l for GPBB and 4.50 μg/l for H-FABP, no study reported significant elevations in H-FABP, and only one found a correlation between GPBB elevation and LV diastolic dysfunction via impaired relaxation (114). However, in a cohort of non-Hodgkin lymphoma subjects treated with doxorubicin-based chemotherapy, H-FABP measured 23 h after the first cycle of CHOP was correlated with LVEF assessed after six cycles (*r* = −0.836, *p* < 0.001) (96). Though numerous studies have found elevated GPBB after chemotherapy, and one has related H-FABP with subsequent systolic dysfunction, none have yet linked biomarker elevations with clinical outcomes in larger populations, which leaves the clinical relevance of these two ischemic markers unclear.

C-reactive protein is an acute phase protein that is synthesized during an inflammatory response. Its expression is regulated by cytokines such interleukin (IL)-1, IL-6, and tissue necrosis factor-α (TNF-α). In the context of stable coronary artery disease, myocardial infarction, and congestive heart failure, elevated CRP is predictive of decreased LVEF and diastolic dysfunction (117–119). Using a high-sensitivity (hs) assay in breast cancer patients, hsCRP concentrations ≥3 mg/l predicted impaired LVEF with 92.9% sensitivity and 45.7% specificity (PPV, 40.6%; NPV, 94.1%). As maximum hsCRP elevations were seen on average 78 days before echocardiographic detection, hsCRP may prove to be effective in identifying patients who are less likely to benefit from more stringent follow-up. While Lipshultz et al. found higher CRP values in survivors of various childhood cancers, regardless of exposure to cardiotoxic treatment with modest correlation with LV mass, wall thickness, and dimension (120), multiple studies have found no clinical value in CRP measurements (79, 84, 89).

Myeloperoxidase is a proinflammatory enzyme that expressed by polymorphonuclear neutrophils that is indicative of oxidative stress, and involved in lipid peroxidation. It has also been identified for its prognostic value in predicting future cardiovascular events in acute coronary syndromes and adverse outcomes in heart failure (121, 122). MPO was identified as one of two predictors of cardiotoxicity in breast cancer patients treated with ACs and Herceptin, from a panel of potential biomarkers including CRP, NT-proBNP, growth differentiation factor (GDF)-15, placenta growth factor (PlGF), soluble fms-like tyrosine kinase receptor (sFlt)-1, and galectin (gal)-3 (89). Ky et al. found that for patients with 90th percentile MPO interval change from baseline (422.6 pmol/l increase), the probability of CREC cardiotoxicity at 15 months was 34.2%, and the risk of future cardiac toxicity was amplified with each standard deviation increase in MPO concentration (HR 1.34, *p* = 0.048). When considered jointly with 90th percentile interval TnI elevations, the risk of cardiotoxicity by 15 months was 46.5%.

Nitric oxide is a small molecule generated by NO synthase from l-arginine in numerous cell types, including endothelial cells, platelets, neutrophils, and macrophage (123). NO is a key regulator of cardiomyocyte contractility, and inducible NO synthase has been implicated in the pathophysiology of heart failure and cardiomyopathy (124, 125). Dysregulated NO synthesis has been found to be one mechanism involved in doxorubicin-induced cardiotoxicity, as studies in bovine endothelial cells have linked redox activation of doxorubicin with endothelial NO synthesis in doxorubicin-induced apoptosis (126, 127). NO has been described as a potential marker of subclinical cardiac dysfunction in the pediatric setting. Guler et al. found significantly higher nitrite values in children treated with doxorubicin compared to healthy controls, and in those with abnormal/borderline LVEF and FS values (92.35 vs. 59.26 μmol/l, *p* = 0.038) (128).

## Conclusion and Future Directions

Cardiac toxicity associated with cancer treatment is a growing source of significant morbidity and mortality. Current screening practices are suboptimal as they provided limited opportunity to intervene and change the course of disease progression. Serum biomarkers, and especially cardiac troponins in patients treated with HDC, represent an effective method for monitoring cardiac status, and identifying patients who may benefit from early medical intervention. There is also growing evidence for a combined approach in which biomarkers and echocardiograms are co-interpreted.

A discussion of any screening test’s validity would be incomplete without considering Wilson and Junger’s classic screening criteria (129). Of the 10 criteria, some are evident, such as “the condition sought should be an important health problem.” And of the 10, the two that deserve additional mention here are “there should be an accepted treatment for patients with recognized disease,” and “there should be an agreed policy on whom to treat as patients.” Both of these questions were addressed by a large randomized study that evaluated the cardioprotective effects of enalapril, an angiotensin-converting-enzyme inhibitor routinely used for congestive heart failure (130). Of 413 patients treated with high-dose ACs in the study, 114 patients developed early increases in TnI and were randomized to receive either enalapril (*n* = 56) or placebo (*n* = 58). In the intervention arm, enalapril was given for 1 year, starting 1 month after chemotherapy. The placebo arm suffered from a significant and progressive decline in LVEF (62.4 vs. 48.3% at 12 months, *p* < 0.001), as well as increases in end-diastolic and end-systolic volume. Moreover, the treatment group benefited from a lower incidence of adverse cardiac events (2 vs. 52%, *p* < 0.001). Other investigators have evaluated the beta-blockers nebivolol and carvedilol in the randomized setting, finding treatment during AC chemotherapy offered significant protection of LVEF in both interventions (131, 132). Though investigations are still ongoing, the results accumulated so far suggests cardiotoxicity, if detected early enough, and treated appropriately, is a potentially treatable condition. Additionally, the study populations and criteria used for treatment have provided a foundation for management decisions that can further refined.

As data on the treatment of chemotherapy-induced cardiotoxicity continue to accumulate, the objective of validating and refining biomarker-based screening strategies becomes more and more clear. Because, clinically apparent signs of cardiac injury often occur years after initial therapy, there are few studies that have been able to link early rises in biomarker concentrations with clinical endpoints. Thus, there is a need longer for long-term data to either confirm or refute any meaningful relationship between early biomarker status and long-term cardiac morbidity. Additionally, because the optimal schedule of biomarker assessments remains unclear, the integration of biomarker evaluations into large prospective clinical trials is critical. As the burden of antineoplastic therapy-induced cardiac morbidity increases, so does the need to find effective strategies for risk stratification and management of therapy-induced cardiotoxicity.

## Conflict of Interest Statement

The authors declare that the research was conducted in the absence of any commercial or financial relationships that could be construed as a potential conflict of interest.
